# A Systematic Review and Meta-Analysis on Microsurgical Safety and Efficacy of Profunda Artery Perforator Flap in Breast Reconstruction

**DOI:** 10.1155/2019/9506720

**Published:** 2019-07-29

**Authors:** Bei Qian, Lingyun Xiong, Jialun Li, Yang Sun, Jiaming Sun, Nengqiang Guo, Zhenxing Wang

**Affiliations:** ^1^Department of Plastic Surgery, Union Hospital, Tongji Medical College, Huazhong University of Science and Technology, 1277 Jiefang Avenue, Wuhan 430022, China; ^2^Department of Medical Records Management and Statistics, Union Hospital, Tongji Medical College, Huazhong University of Science and Technology, 1277 Jiefang Avenue, Wuhan 430022, China

## Abstract

**Background:**

The profunda artery perforator (PAP) flap was first applied in breast reconstruction in 2010 by Robert J. Allen. It provided an alternative for autologous breast reconstruction in addition to traditional donor sites. Currently, literature reporting its microsurgical safety and efficacy is relatively sparse and heterogeneous*. Objective*. To clarify the evidence regarding microsurgical safety and efficacy of PAP flap in breast reconstruction, which may contribute to future surgical decision-making.

**Methods:**

Multiple databases were systematically searched by two independent reviewers. The result was statistically analyzed with Meta command of R GUI 3.5.1. The proportions with 95% confidence intervals (CIs) were calculated by using random-effect model.

**Results:**

There were 12 studies including 516 PAP flaps meeting the inclusion criteria. The pooled surgical success rate was 99% (95% CI: 97%–100%) and overall rate of complications was 23% (95% CI: 18%–27%). The most common individual complication was wound dehiscence with incidence of 6% (95% CI: 4%–9%). The seroma rate was 2% (95%CI: 0%–6%). The hematoma rate was 1% (95% CI: 0%–2%). The partial necrosis rate was 2% (95% CI: 0%–5%). The rate of total flap loss was 1% (95% CI: 0%–3%).

**Conclusion:**

To date, this study is the first meta-analysis of microsurgical efficacy and safety evaluation of the PAP flap in breast reconstruction. This present work confirmed that the PAP flap is safe and reliable in breast reconstruction with high success rate, but a relatively low complication rate. Moreover, it might be more than an alternative to the deep inferior epigastric perforator flap (DIEP) in microsurgical breast reconstruction in selected patients.

## 1. Introduction

The autologous breast reconstruction is playing an increasingly significant role in the comprehensive treatment of breast cancer due to long-term psychosexual health and its importance for breast cancer survivors [1]. It is recommended to all patients with breast cancer by the National Institute for Health and Clinical Excellence (NICE), UK [2]. And in fact, postmastectomy breast reconstruction continues to experience an upward trend [3]. Although both autologous tissue-based and implant-based reconstruction provide satisfactory reconstructive results, autologous tissue breast reconstruction is often considered to be superior to implant-based breast reconstruction in creating a natural breast mound and maintenance of long-term aesthetic results [1, 4]. Many previous literature works have shown that the DIEP derived from the abdomen is the first choice for autologous breast reconstruction [5–7]. However, sometimes abdominal tissue is not a suitable option for patients with a history of abdominal surgery or insufficient available volume, or it is just a preference of patients to avoid an abdominal scar [8–10]. The diversity of patients prompts plastic surgeons to seek flaps from other regions of bodies for breast reconstruction, for example, superior or inferior gluteal artery perforator flap (SGAP/IGAP); transverse, vertical, or diagonal upper gracilis flap (TUG/VUG/DUG); lateral thigh perforator flap (LTP); anterolateral thigh flap (ALTP); and transverse musculocutaneous gracilis (TMG) flap [11–17]. Actually, the posterior thigh region has been neglected as a potential donor site for breast reconstruction [18]. The PAP flap is a variation of the posterior thigh myocutaneous flap. It was firstly described by Hurwitz and Walton in 1980 [19] and then modified by Angrigiani et al. [20, 21]. As a free flap, the PAP flap has also been used for burn and pressure sore reconstruction by Song et al. [22]. In 2012, the PAP flap was firstly introduced as an alternative to the DIEP flap for breast reconstruction by Robert J. Allen et al. [23] and then quickly emerged as a second choice for autologous tissue breast reconstruction [24–27]. Currently, literature reporting the microsurgical safety and efficacy of the PAP flap in breast reconstruction is relatively rare and heterogeneous. Thus a systematic review of its microsurgical performance was needed. We conducted a meta-analysis on microsurgical complication and safety of the PAP flap in breast reconstruction. This work aimed to provide a relatively reliable evidence on whether the PAP flap was an ideal alternative for autologous breast reconstruction.

## 2. Materials and Method

### 2.1. Literature Search

Chinese and English databases, including China National Knowledge Infrastructure (CNKI), Wan Fang Data Knowledge Service Platform (Wan Fang), PubMed, EMBASE, and Cochrane Library, were searched for articles from January 2010 to September 2018 according to the Preferred Reporting Items for Systematic Reviews and Meta-Analyses checklist [28]. The search strategy combining MeSH keywords with free words “((Profunda Artery Perforator Flap or PAP [All Fields])) AND (Breast Reconstruction or Breast Reconstructions or Reconstruction, Breast or Reconstructions, Breast or Mammaplasties or Mammoplasty or Mammoplasties [All Fields])” was used. We also complemented electronic retrieval by scanning selected articles and references from other sources. Also, there was no language restriction.

### 2.2. Selection Criteria

The inclusion criteria for this review consisted of the following: (1) clinical studies; (2) consecutive cases; (3) the PAP flap transfer as the surgical method; (4) breast reconstruction as major object; (5) availability of clinical data of postoperative complications; (6) containing more than two cases. The exclusion criteria were as follows: (1) reviews, abstracts, or letters; (2) studies with ambiguous results; (4) out-and-out anatomical basis or surgical method of PAP flap; (5) articles that could not be accessed. If the duration and sources of study population recruitment overlapped in two or more articles by the same authors, only the most recent study or the study with the larger number of participants was included.

### 2.3. Data Extraction

Two investigators (B.Q. and L.y.X.) scrutinized all the potential relevant articles to identify eligible studies using predefined inclusion and exclusion criteria. Disagreements between the two investigators were resolved by reassessment of the original article by a third investigator (senior author) and discussion on evidence. Data were extracted from the included studies. And then all the data were counted in a spreadsheet that included column names of the authors, publication date, country of origin, mean body mass index (BMI), sample size of patient and flap, average flap weight and pedicle length, overall postoperative complication, and flap loss cases. Postoperative complications included hematoma, seroma, partial necrosis, and donor-site wound dehiscence. The quality of each included study was assessed according to the Newcastle–Ottawa scale (NOS) [29], which was a risk assessment tool for nonrandomized studies in a meta-analysis.

### 2.4. Statistical Analysis

The data-processing software program R GUI 3.5.1 (the R foundation for statistical computing, the United States) was used for the statistical analysis of the included studies. Summary results were shown with an incidence rate of the events (ratio of event number to patient number) and proportions with 95%CI. The statistical heterogeneity among the included studies was evaluated using I^2^ statistics and Q statistic P values. It measured the percentage of total variation across studies. I^2^> 50% or P<0.05 was regarded as significant heterogeneity. Random-effects model was used in all analyses to cover the variation between and within studies [30]. The Meta command (meta-analysis of single proportions) was applied to evaluate the complication rates and corresponding CIs. Arcsine transformation was set as the summary measure [31]. If any study had a zero or one cell count, a continuity correction was applied. Then the forest plot was applied to illustrate the pooled results and funnel plot was used to look for evidence of publication bias [32]. Moreover, the asymmetry was tested by Egger's linear regression approach [33].

## 3. Result

### 3.1. Literature Search

**Figure 1:**
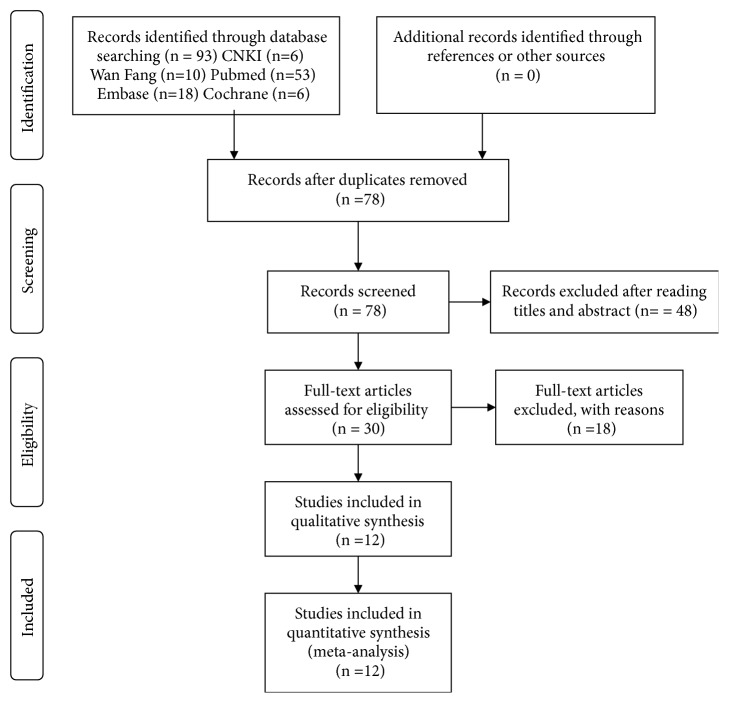
Flow diagram of the literature search and selection. CNKI: China National Knowledge Infrastructure.

Through screening English databases, 77 potentially relevant publications were initially identified, including 53 from PubMed, 18 from EMBASE and 6 from Cochrane Library. The Chinese database retrieved 16 articles: 10 articles from Wan Fang and 6 from CNKI. There were 93 literature works included in total. 78 articles remained after duplicate search results were removed. 48 studies were excluded by scanning titles and abstracts, and then just 30 studies remained. All the remaining literature works were carefully read, including references, to make sure they meet primary search criteria. And then 18 studies that did not meet the inclusion criteria were deleted. Ultimately, 12 studies were selected for this present meta-analysis. A flow diagram of the literature selection process was shown in Figure 1.

### 3.2. Clinical Characteristics of Included Studies

Among the 12 studies finally identified, 516 PAP flaps were included and their characteristics are shown in Table 1 [24, 27, 34–43]. They were all published in the past four years, four studies in the United States of America, three in France, three in China, and two, respectively, in the United Kingdom and the Netherlands. Statistics on various complications are shown in Table 2. The column of “other complications” includes local sensory disturbance, anastomotic arterial and venous thrombosis, lymphedema, local cellulitis, leg compartment syndrome, and wound retraction. Because they were not widely reported, there were no corresponding analyses here. However, the emergence of these complications should be seriously taken into account during the perioperative management of patients. The included studies were all retrospective with a low evidence level. Therefore, performing further statistical analysis based on these data was necessary.

### 3.3. Quality of Included Studies

The NOS score ranged from 5 to 6 by carefully analyzing the quality of each included study in strict accordance with the NOS scoring criteria. The results were shown in Table 1. The overall quality of them was medium (maximum score=9).

### 3.4. Pooled Surgical Success Rate

**Figure 2:**
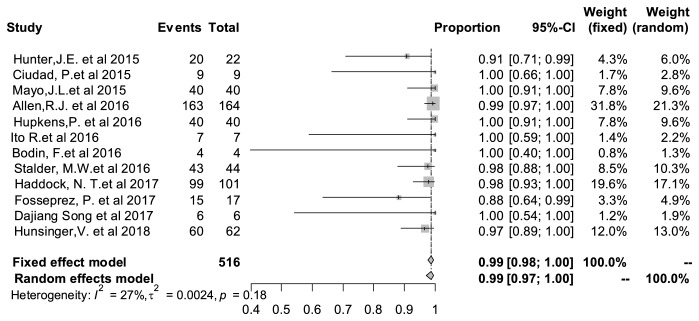
Forest plot of pooled surgical success rate. The marker size represented the weight of all included studies.

As shown in Figure 2, the pooled surgical success rate was 99% (95% CI: 97%–100%). It confirmed the effects and safety of this surgical approach to some extent. Literature heterogeneity testing showed I^2^* *=* *27% and P=0.18, indicating the highly diverse literature.

### 3.5. Overall Rate of Complication

**Figure 3:**
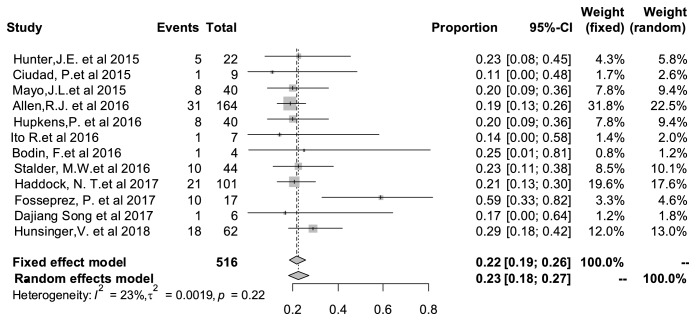
Forest plot of overall complications rate after surgery. The marker size represented the weight of all included studies.

The incident rate of overall complication was shown in Figure 3. Among the 12 studies reporting complications, there was a pooled complication rate of 23% (95%CI: 18%–27%). And there was no significant statistical heterogeneity among the studies.

### 3.6. Wound Dehiscence Rate

**Figure 4:**
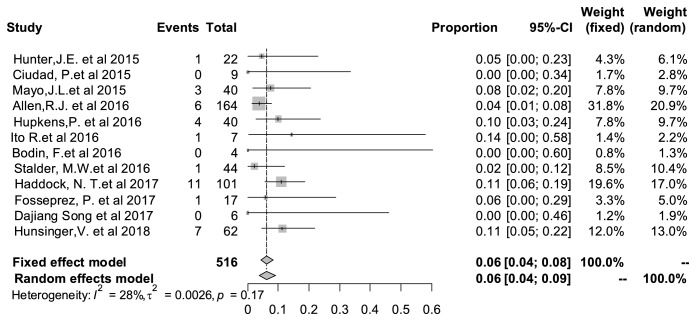
Forest plot of pooled wound dehiscence rate after surgery. The marker size represented the weight of all included studies.

The wound dehiscence was relatively more common among various complications. In Figure 4, it was demonstrated that the rate of this complication was 6% (95% CI: 4%–9%) Heterogeneity test indicated that I^2^* *=* *28% and P=0.17, which showed no significant statistical heterogeneity among the studies.

### 3.7. Seroma Rate

**Figure 5:**
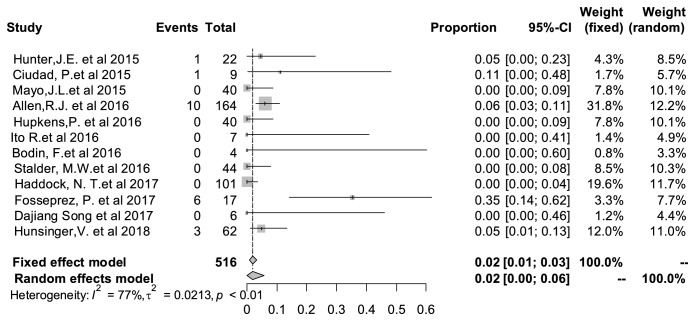
Forest plot of pooled seroma rate after surgery. The marker size represented the weight of all included studies.

Like most surgical approaches of autologous tissue breast reconstruction, seroma also occurred in the PAP flap during postoperative period. The rate of the complication was 2% (95% CI: 0%–6%), shown in Figure 5. Heterogeneity test implied that I^2^=* *77% and P<0.01, indicating significant statistical heterogeneity between the studies.

### 3.8. Hematoma Rate

**Figure 6:**
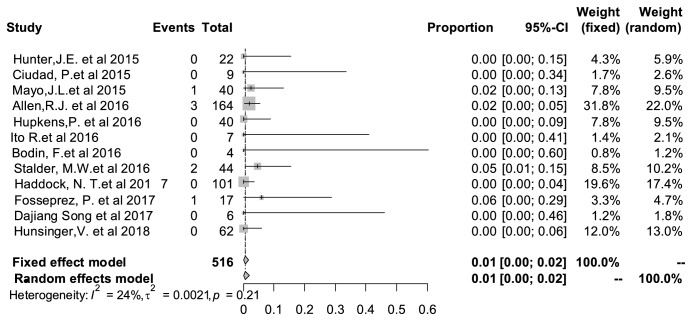
Forest plot of pooled hematoma rate after surgery. The marker size represented the weight of all included studies.

From the statistical results, the incidence of hematoma was lower than seroma.  Figure 6 showed that the pooled complication rate was 1% (95% CI: 0%–2%). No significant heterogeneity was observed as well (I^2^=* *24%, P=0.21).

### 3.9. Partial Necrosis Rate

**Figure 7:**
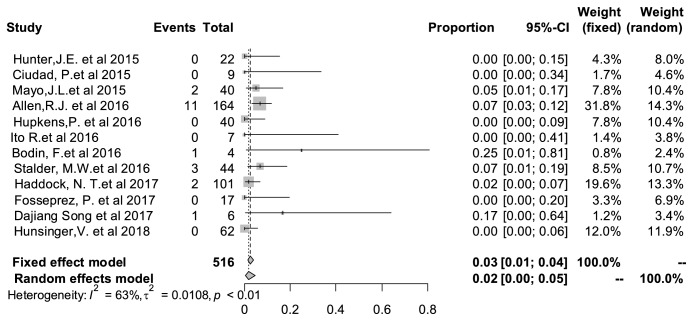
Forest plot of pooled partial necrosis rate after surgery. The marker size represented the weight of all included studies.

Necrosis reflects the blood supply of the flap after reconstruction operation. The partial necrosis rate of PAP flap in breast reconstruction was 2% (95% CI: 0%–5%), shown in Figure 7. Heterogeneity test indicated that I^2^=* *63% and P<0.01, which meant a significant statistical heterogeneity here.

### 3.10. Total Flap Loss Rate

**Figure 8:**
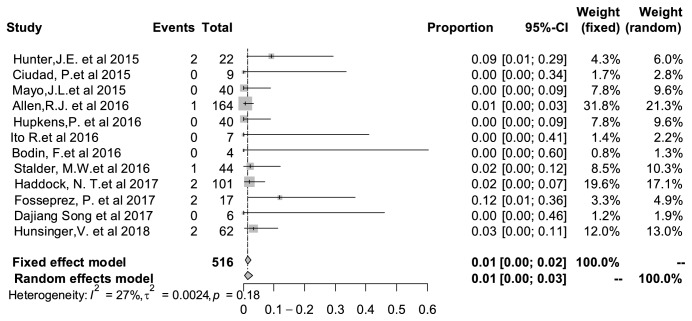
Forest plot of pooled total flap loss rate after surgery. The marker size represented the weight of all included studies.

The rate of total flap loss was 1% (95% CI: 0%–3%), with I^2^ value of 27% and P value of 0.18. There was no statistical heterogeneity among the pooled studies too.  Figure 8 shows detailed results of flap loss rate analysis.

### 3.11. Publication Biases

**Figure 9:**
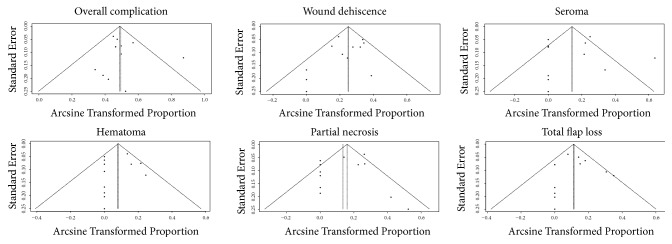
Funnel plot about various complications.

**Figure 10:**
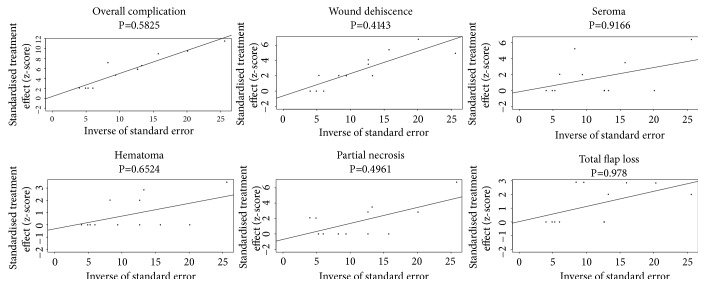
Linear regression test of funnel plot asymmetry.

The funnel plot about various complications after PAP flap breast reconstruction was presented in Figure 9. From the general view of the image, the distribution of each study in the triangle was relatively symmetrical. Surely, we also used the asymmetry test with Egger's linear regression to evaluate and verify the statistical symmetry of the funnel plot by calculating the P value. The results were shown in Figure 10. P < 0.05 was regarded as significant publication bias. The results revealed that the funnel plot was statistically symmetrical, which meant no significant publication bias in the study.

## 4. Discussion

Since the PAP flap was firstly applied in breast reconstruction, it has become increasingly popular and a preference of many surgeons and patients. However, with this growing trend, reports of complications have surfaced. It suggested the need for a systematic review and meta-analysis of the PAP flap to evaluate its microsurgical safety and efficacy in breast reconstruction. To our knowledge, this study was the first to evaluate microsurgical safety and efficacy of the PAP flap in breast reconstruction. Compared with previous studies, a higher level of evidence was provided. We initially established the PAP flap as a safe and feasible approach in autologous breast reconstruction, which offered postmastectomy patients an reliable alternative for autologous breast reconstruction in addition to traditional flaps, especially when the abdominal tissue is not indicated.

According to the included literature, the microsurgical performance of the PAP flap in breast reconstruction could be concluded in the following three aspects: (1) The PAP flap provided a relatively large amount of skin and subcutaneous soft tissue that could be easily shaped. Among the selected studies, Allen RJ et al. reported a mean flap weight of 366g [24] and Haddock NT et al. showed 425g [41]. With proper patient choice, a sufficient breast volume could be obtained. In addition, natural-shaped breasts and stable long-term reconstructive outcome could be successfully achieved in most cases. (2) The PAP flap had relatively constant vascular anatomy. A long pedicle with sufficient vessel diameter made it an excellent match for the thoracic recipient vessels. In the included literature, Allen RJ et al. reported an average pedicle length of 10.2 cm, an arterial diameter of 1.7 mm, and a vein diameter of 2.6 mm [24]. DeLong MR et al. reported the presence of at least two profunda perforators in each thigh [44]. Saad A et al. demonstrated that the perforators could be consistently found in the posterior thigh region [45]. The feasibility of microsurgical anastomosis was comparable to the DIEP flap, and the learning curve length of flap harvesting was acceptable. (3) The PAP flap had excellent donor-site cosmetics. Most included studies showed that the donor-site scar was coincidentally hidden in the gluteal fold [23] and the damage to the function and contour of the thigh was minimized due to muscle sparing. In summary, these excellent performances made it theoretically an ideal flap for breast reconstruction.

Based on our statistical results, it could be demonstrated that the PAP flap was a safe and reliable option for breast reconstruction. The argument included the following three points: high success rate, low complication rate, and satisfactory aesthetic outcome. Firstly, the pooled surgical success rate was 99% (95% CI: 97%–100%), which was comparable to that of the DIEP flap. Gill PS et al. reported that a total flap loss rate in all their 758 DIEP flaps was 0.5% [46]. Knox ADC et al. indicated that the rate of DIEP was 1.1% [47]. The high success rate suggested that the surgical technique was feasible in either the harvesting of the flap or the anastomosis of the blood vessels. Secondly, the overall rate of complications was just 23% (95% CI: 18%–27%). Various complications were summarized in Table 2. The result was almost equal to that of the DIEP flap (Ochoa O et al. reported that the rate of DIEP was 23.8% [48]) and less than muscle-sparing transverse rectus abdominis myocutaneous (MS-TRAM) or superficial inferior epigastric artery (SIEA, Wang X-L et al. reported that the rates of MS-TRAM and SIEA were 25.6% and 26.7%, respectively [49]). Thirdly, almost all included studies showed a satisfactory aesthetic outcome of the donor site, such as minimal contour deformity or invisible incision scar [24, 27, 34, 41]. All in all, the PAP flap not only created a natural, permanent, and autologous tissue breast, but also ensured good donor-site aesthetics and low complication rate.

As expected, the partial necrosis rate and total flap loss rate were just 2% (95% CI: 0%–5%) and 1% (95% CI: 0%–3%), respectively. It implied a reliable blood supply of PAP flap. Although Allen RJ et al. reported one flap loss, it was because of a technical error in perforator isolation [24]. The result may also benefit from a multiteam approach which saved operative time and shortened potential flap ischemia time. In this point, Haddock N et al. indicated that flap loss rate of PAP was in line with reported failure rates for the DIEP flaps [26]. Furthermore, we noticed that the anastomotic arterial and venous thrombosis have not been widely reported in all included literature, which meant that the pedicle length and caliber of PAP flap exactly matched up with vessels of the recipient. The result was consistent with the reported literature [24, 41]. The incidence of wound dehiscence was more common with a rate of 6% (95% CI: 4%–9%), which was almost equal to the DIEP flap [47, 48] (Knox ADC et al. reported that the rate was 6%, and Ochoa O et al. reported that the rate was 6.2%) and less than the TUG flap [17] (Schoeller T et al. indicated that the rate was 7.3%). Just as the literature reported, maybe a semicircular incision or postoperative supine position or sitting position disturbed normal wound healing. Therefore, a comprehensive preparation of wound and a more individual postoperative care were essential to provide a comparable surgical outcome [24]. Haddock NT et al. reported that incisional wound vacuum dressings and compression garments might decrease surgical-site complications [41]. Since no muscle was sacrificed in the PAP flap comparing to the TUG flap, less seroma accumulation occurred [34]. Buntic RF et al. reported a seroma rate of 15.6% after harvesting of the TUG flap [50], while our pooled seroma rate of the PAP flap was just 2% (95%CI: 0%–6%), which was obviously lower than TUG flap. Besides, the mean body mass index of patients from included studies was 23.52kg/m^2^ and the average flap weight was 360g. This result suggested that the PAP flap could be sufficient for reconstruction of total mastectomy defects in selected patients with small to moderate sized breasts. As for big breast size, Mayo JL et al. stacked the PAP flap and the DIEP flap [36]. Allen RJ et al. used autologous fat grafting and local flaps to augment the breast [24].

Compared with traditional flaps, we tentatively put forward three advantages of PAP flap. One of the strongest advantages of the PAP flap is bilateral reconstruction. In small to moderate sized breasts, we suggest that it should be the first choice. Although it is possible to split a DIEP flap for bilateral reconstructions [51, 52], there are always some tradeoffs. Enough tissue can be harvested redundantly for both sides from posterior thigh region with a very symmetrical and inconspicuous donor-site contour. Moreover, compared to other lower limb donor flaps such as TMP flap and TUG flap, there is no muscle sacrificed in the PAP flap, which means less seroma accumulation and less donor-site contour and functional damage. Secondly, when it comes to scars, the PAP flap might even be superior to any abdominal donor-site flaps. As the scar is perfectly hidden under the groin and gluteal fold even though the patient is entirely naked. Thirdly, young women within a certain age range typically do not show redundant abdominal tissue. However, they present with enough tissue on the posterior thigh region for reconstruction of small to moderate breasts.

In view of the statistics, it was found that heterogeneities of pooled hematoma, total flap loss, partial necrosis, and wound dehiscence were very low (I^2^ value<50%; Q statistics P>0.05). Significant heterogeneity was found in pooled seroma rate and partial necrosis rate of this study. Heterogeneity commonly existed among case series studies, due to variations in patients' characteristics, different experiences of surgeons, and various surgical techniques, as well as nonrandomized study designs. The random-effects model was employed to minimize the impact of heterogeneity on the stability of pooled results. We intended to detect the reason for these heterogeneities; unfortunately, due to the above-mentioned limitation in available data, subgroup analysis was challenging to implement and subsequently did not reveal the origin of heterogeneity. During the review of publications, some common shortcomings were observed. Only a few studies have provided relevant clinical data on perioperative risk factors and operational details that may affect surgical outcomes, such as wound sizes, the choice of recipient vessels, and the type of vascular anastomosis. Therefore, a meta-regression on potential risk factors has not been performed. These above-mentioned weak points should be covered by future more large samples and multicenter researches with standardized parameters and endpoints, to promote the scientific quality as well as provide more useful information to a future meta-analysis.

## 5. Limitations

There were several limitations in this study. The relatively small sample size weakened the strength of evidence to some extent. Also, long-term evaluation of end result and BREAST-Q evaluations were absent, which were important factors evaluating the PAP flap. More studies with large cohort and randomized design for a closer estimation are needed in the future.

## 6. Conclusion

The PAP flap was a safe and reliable option for breast reconstruction with a high success rate, but a low complication rate. Moreover, it might be more than an alternative to the DIEP flap in microsurgical breast reconstruction in selected patients, such as bilateral breast reconstructions, or preference of patients to avoid an abdominal scar or young women without redundant abdominal tissue. Additionally, more large samples and multicenter researches with standardized reports of perioperative parameters and clinical outcomes are needed for further evaluation in the future.

## Figures and Tables

**Table 1:** Clinical characteristics of included studies. NOS: Newcastle–Ottawa scale. BMI: body mass index.

							Sample size		Mean		No. of	
Study	Author	Year	Origin	Reference	NOS	BMI(kg/m^2^)	Patient	Flap	Flap weight(g)	Pedicle length(cm)	Complications	Failures
1	Hunter, J. E.et al	2015	UK	[34]	6	21.6	13	22	242	9.9	5	2
2	Ciudad, P.et al	2015	China	[35]	5	22.4	9	9	466.1	9.25	1	0
3	Mayo, J. L. et al	2015	USA	[36]	5	23.7	20	40	299	/	8	0
4	Allen, R. J. et al	2016	USA	[23]	6	22.5	96	164	367.4	10.2	31	1
5	Hupkens, P. et al	2016	NED	[37]	6	23.3	30	40	372.4	11	8	0
6	Ito R.et al	2016	China	[38]	5	23.5	5	7	257.1	9.4	1	0
7	Bodin, F.et al	2016	France	[39]	5	21.7	4	4	303	9	1	0
8	Stalder, M.W.et al	2016	USA	[40]	6	24.8	22	44	/	/	10	1
9	Haddock, N. T.et al	2017	USA	[41]	6	26.8	56	101	425	10.3	21	2
10	Fosseprez, P. et al	2017	France	[27]	5	22.73	15	17	/	/	8	2
11	Dajiang Song et al	2017	China	[42]	5	/	6	6	345	7.2	1	0
12	Hunsinger,V. et al	2018	France	[43]	6	23.6	51	62	309.4	10.7	18	2

**Table 2:** Statistics about various complications. Others include local sensory disturbance, anastomotic arterial and venous thrombosis, lymphedema, local cellulitis, leg compartment syndrome, and wound retraction.

Study	Author	Total complications	Flap loss	Wound dehiscence	Partial necrosis	Seroma	Hematoma	Others
1	Hunter, J. E.et al	5	2	1	0	1	0	1
2	Ciudad, P.et al	1	0	0	0	1	0	0
3	Mayo, J. L. et al	8	0	3	2	0	1	2
4	Allen, R. J. et al	31	1	6	11	10	3	0
5	Hupkens, P. et al	8	0	4	0	0	0	4
6	Ito R.et al	1	0	1	0	0	0	0
7	Bodin, F.et al	1	0	0	1	0	0	0
8	Stalder, M.W.et al	10	1	1	3	0	2	3
9	Haddock, N. T.et al	21	2	11	2	0	0	6
10	Fosseprez, P. et al	8	2	1	0	6	1	0
11	Dajiang Song et al	1	0	0	1	0	0	0
12	Hunsinger,V. et al	18	2	7	0	3	0	6
